# Appreciation and coping strategies adopted by children hospitalized for COVID-19 and their primary caregivers

**DOI:** 10.1590/0034-7167-2025-0265

**Published:** 2026-07-31

**Authors:** Bárbara Radieddine Guimarães, Juliana Barony da Silva, Nayara Luiza Henriques, Ana Clara Gomes Andrade, Luiza Araujo dos Santos, Paulo Roberto Lima Falcão do Vale, Elysângela Dittz Duarte

**Affiliations:** I Universidade Federal de Minas Gerais. Belo Horizonte, Minas Gerais, Brazil; II Universidade Federal do Recôncavo da Bahia. Cruz das Almas, Bahia, Brazil

**Keywords:** COVID-19, Nursing, Child, Caregivers, Coping Skills., COVID-19, Enfermería, Niño, Cuidadores, Habilidades de Afrontamiento.

## Abstract

**Objectives:**

to analyze appreciation and coping strategies of children hospitalized for COVID-19 and their primary caregivers during the illness and hospitalization process.

**Methods:**

a qualitative and exploratory study, based on the McCubbin & McCubbin model (1993), with 25 participants: ten children hospitalized for COVID-19 and 15 primary caregivers. A semi-structured interview was used, and data were subjected to reflective thematic analysis.

**Results:**

children reported a predominantly negative appreciation of hospitalization, marked by fear, homesickness, pain, and discomfort with hospital routine. Their coping strategies included play, electronic devices, hygiene, and adherence to treatment. Caregivers also expressed negative assessments related to fear of death, isolation, and distress. Their strategies involved spirituality, religiosity, love for their children, and support from professionals and family members.

**Final Considerations:**

coping strategies differed between children and caregivers, highlighting the importance of care sensitive to the dyad’s needs.

## RESUMEN

**Objetivos:**

analizar la apreciación y estrategias de afrontamiento de niños hospitalizados por COVID-19 y sus cuidadores principales durante la enfermedad y el proceso de hospitalización.

**Métodos:**

estudio cualitativo y exploratorio, basado en el modelo de McCubbin y McCubbin (1993), con 25 participantes: diez niños hospitalizados por COVID-19 y 15 cuidadores principales. Se utilizó una entrevista semiestructurada y los datos se sometieron a un análisis temático reflexivo.

**Resultados:**

los niños manifestaron una apreciación predominantemente negativa de la hospitalización, caracterizada por miedo, nostalgia, dolor e incomodidad con la rutina hospitalaria. Sus estrategias de afrontamiento incluían juego, dispositivos electrónicos, higiene y adherencia al tratamiento. Los cuidadores también expresaron evaluaciones negativas relacionadas con el miedo a la muerte, el aislamiento y la angustia. Sus estrategias incluyeron la espiritualidad, la religiosidad, el amor a sus hijos y el apoyo de profesionales y familiares.

**Consideraciones Finales:**

las estrategias de afrontamiento difirieron entre los niños y sus cuidadores, lo que resalta la importancia de una atención sensible a las necesidades de la díada.

## INTRODUCTION

The COVID-19 pandemic, which began in 2019, had a global impact on social, economic, and public health, causing thousands of deaths. Children aged 0 to 14 years represented between 1.68% and 16.34% of cases between December 2019 and July 2023^(1)^. Epidemiological data have indicated less severe health outcomes for this child population compared to the adult population^(2)^; however, there are records of severe manifestations and the need for pediatric hospitalizations due to this cause^(3)^.

Hospitalization is a stressful event for the whole family, causing changes in the routine and well-being of its members. When children are hospitalized, the physical and emotional dependence inherent in this group brings additional demands to caregivers and family members, who report burnout related to altered routine, increased expenses, and sleep deprivation^(4)^. Furthermore, feelings of distress, anxiety, loss of privacy, and worry have been identified in children and their caregivers in hospital settings^(4,5)^. Pain and fear, in turn, are experiences reported only by children^(4,5)^.

In the context of the COVID-19 pandemic, the hospitalization experience had particularities, such as the possibility of contamination of family members and the stigmatization of the disease^(6,7)^, especially before the availability of vaccines and the recognition of the greater severity of cases that required hospitalization^(6)^. These aspects have been identified as contributing to accentuating children’s vulnerability, generating worry and stress^(6)^, and aggravating anxiety and depression in the entire population^(7)^.

Even with common aspects, the way of dealing with stressful situations, such as the hospitalization of a child due to COVID-19, varies according to family dynamics and the adaptations made by each member and the family as a whole^(8)^. Therefore, the way families perceive the same stressful event-in this study, a child’s illness and hospitalization-can vary among families and their members, as can the resources they use to adapt to this situation^(8)^.

In this study, the theoretical Resiliency Model of Family Stress, Adjustment, and Adaptation^(8)^ and its definition of appreciation and coping were adopted. Appreciation is defined as the way in which a family and/or its members, individually, perceive and feel a stressful event, its severity and the meanings of changes that occurred in family dynamics and daily life^(8)^. Coping, in turn, refers to the strategies, efforts, and behaviors adopted by a family and/or its members individually to maintain or restore the balance between family demands and resources, reducing the severity of the stressor-in this study, hospitalization due to COVID-19 and its challenges^(8)^.

Therefore, a family’s assessment of the situation and coping strategies are interrelated and can be modified as families become more vulnerable due to an accumulation of demands when a member becomes ill^(8)^.

It is noteworthy that, depending on each family member’s assessment of the situation, coping efforts may vary. Therefore, it is important to explore, from the perspective of children and primary caregivers, the coping strategies used during hospitalization for COVID-19.

## OBJECTIVES

To analyze appreciation and coping strategies of children hospitalized for COVID-19 and their primary caregivers during the illness and hospitalization process.

## METHODS

### Ethical aspects

The research was approved by the *Universidade Federal de Minas Gerais* Research Ethics Committee and by the Municipal Health Department of Belo Horizonte, in accordance with Resolutions 466/12 and 580/18 of the Brazilian National Health Council. Participation was authorized through the signing of an Informed Consent Form by caregivers and an Informed Assent Form by children, sent electronically or by mail.

### Theoretical and methodological framework

The research was guided by the Resiliency Model of Family Stress, Adjustment, and Adaptation^(8)^, which allowed us to understand the hospitalization of a child with COVID-19 as a stressful moment experienced by the family, in which strategies can be adopted to build a more positive perspective.

### Study design

This is a qualitative exploratory study, whose elaboration followed Consolidated criteria for Reporting Qualitative research recommendations^(9)^.

### Methodological procedures

The families in the study were identified from a database made available by the epidemiological surveillance of the municipality of Belo Horizonte, Minas Gerais. This database gathered information from the notification forms of 83 children who had been hospitalized for COVID-19.

Based on this data, initial telephone contact was made with the families to present the study objectives and invite primary caregivers to participate. Upon acceptance, the best day and time was scheduled, according to family members’ availability.

It is noteworthy that, among the 83 children in the provided database, 44 did not have contact information or the number was nonexistent; ten did not answer; seven caregivers reported that a child was not hospitalized for suspected or confirmed COVID-19; and seven refused to participate in the research.

Inclusion criteria comprised primary caregivers who lived in the same household as the children and children aged 6 to 12 years - based on their capacity for understanding and reflection^(10)^ - hospitalized due to suspected or confirmed SARS-CoV-2 infection. Exclusion criteria included caregivers with communication limitations or psychological/psychiatric alterations that made it impossible to understand the investigative procedures.

### Study setting

This study was conducted in the municipality of Belo Horizonte, Minas Gerais. Participant identification was carried out using a database provided by the city’s epidemiological surveillance department.

The study was conducted through video call interviews (WhatsApp^®^ or Google Meet^®^), respecting the conditions imposed by the health context of the period.

### Data source

Study participants were ten dyads composed of children who were hospitalized for COVID-19 and their respective primary caregivers, in addition to five caregivers whose children could not participate for the following reasons: two became ill on the day of the interview; one refused to participate; one was absent at the time of the interview; and one died after hospitalization.

### Data collection and organization

For data collection, a sociodemographic questionnaire was used, applied to primary caregivers, in addition to semi-structured interviews conducted with scripts developed based on the McCubbin & McCubbin model^(8)^, which were different for children and caregivers. The questions were guided by the challenges experienced and coping strategies during hospitalization, including questions such as “What was it like being in the hospital for a while because of the coronavirus?” (child) and “Tell me what it was like for you to receive the news that [name] would need to be hospitalized for COVID?” (caregiver).

The interviews were conducted separately, with caregivers and children being instructed to remain alone to ensure data reliability and freedom of expression. Only in two situations was the child in the same room as the caregiver.

Data collection took place between October 2021 and January 2022, with the interval between hospitalization and the interview ranging from five months to a year and a half. The interviews were audio-recorded and lasted an average of 34 minutes. During the interviews, a field diary was produced, the records of which were used by the researcher to support, in particular, the information analysis.

Contacts were made by one of the authors, a nurse with experience in research and childcare, in the context of her master’s degree.

### Data analysis

The interviews were transcribed, and qualitative data were subjected to reflective thematic analysis^(11)^, guided by the Resiliency Model of Family Stress, Adjustment, and Adaptation (McCubbin & McCubbin, 1993)^(8)^. MAXQDA^®^ was used to manage, code, and explore the data. Codes were constructed from the reference framework used and from common aspects between children’s and caregivers’ statements that could help answer the research question.

Initially, six interviews (three with children and three with primary caregivers) were coded by two independent researchers. Subsequently, codes and any doubts or discrepancies were discussed with a third author. During this coding consensus phase, a Kappa index greater than 0.75 was achieved for inter-coder agreement. Once the interviews were coded, an analytical matrix was constructed containing a synthesis of each participant’s information from each code. The matrix allowed for visibility of individual experiences and the identification of patterns in the data produced.

This process resulted in the definition of the following themes: “Appreciation and coping strategies adopted by children hospitalized for COVID-19” and “Appreciation and coping strategies of primary caregivers regarding the hospitalization of children due to COVID-19”.

## RESULTS

Twenty-five participants were interviewed, comprising 15 primary caregivers and ten children hospitalized for COVID-19, forming ten dyads. Dyads M8, M9, M10, M11, and M12 did not form. Among the caregivers, 13 were mothers and two were grandmothers, aged between 25 and 49 years (median = 42). Most were married (n=9), had some religion (n=13), self-identified as mixed-race (n=9), had paid employment (n=10), and had incomplete higher education (n=6). The ten children were between 6 and 11 years old (median = 6), most were male (n=6), hospitalized in private hospitals (n=8), with a median length of stay of seven days (2-96 days). Four had comorbidities such as asthma (n=3) and seizures (n=1).

Regarding the pandemic context, 11 hospitalizations occurred with Intensive Care Unit occupancy above 70%, and four during periods of greater social restriction. All hospitalizations occurred before the COVID-19 vaccine’s availability for the pediatric group.

To improve the presentation of results, this session was organized into two themes: “Appreciation and coping strategies adopted by children hospitalized for COVID-19” and “Appreciation and coping strategies of primary caregivers regarding the hospitalization of children due to COVID-19”. Patterns of predominantly positive experiences were identified, associated with feelings such as joy, confidence, and tranquility, and predominantly negative experiences, marked by dissatisfaction, difficult feelings, and stressful events experienced during hospitalization.

### Appreciation and coping strategies adopted by children hospitalized for COVID-19

Children’s perceptions of hospitalization due to COVID-19 were expressed through their feelings and the meanings they attributed to illness, symptoms, and the care they received. Data analysis revealed that the coping strategies they adopted were diverse and related to the different forms of appreciation they expressed.

Fear was a feeling mentioned by children as responsible for a predominantly negative assessment, as presented by C2, who said she thought “I was going to die!”. C3 mentions feeling fear after hearing reports of relatives dying: “A relative of mine died because of COVID, so you were scared, right?”.

Besides fear, the “isolation, monotony, and lack of interaction” in the hospital were reasons for predominantly negative assessments by the children. C3, C4, C7, and C13 talk about this when they report that, during their hospitalization, they could not do anything because “being quiet was the only thing I had to do” (C7). They “had to keep looking at the same view all the time [...] they had to stay in the room alone” (C4).

Also contributing to negative assessments was the fact of “not being in their [own] home” (C3). Children reported feelings such as missing “going to my grandma’s house” (C3) and “my family” (C7), and sadness at knowing that “if you’re in the hospital, you know you’re not well” (C3). Some children reported feeling that the hospitalization time was too long: “I was hospitalized there for 4,000 days!” (C6); “the hospital’s smell was even good, but being hospitalized for a thousand years afterwards, no” (C4). For these assessments, maintaining contact with family members through cell phones was a coping strategy. Devices such as televisions and tablets were used to reduce homesickness (C1, C6), fear (C2, C6, C13), sadness (C6), boredom (C7), and to “pass the time” (C3) and “distract” (C1). The use of play was also a strategy used. Children played with their “games” (C2, C13), as well as “animals and dolls” (C13) to “distract themselves a little” (C13) and to feel less “fear” (C2, C13) and “sadness” (C2) in the hospital. In addition to play, spending time with their caregivers and accepting their support were also seen as strategies for managing the tensions related to hospitalization.

Aspects of hospital structure, services, and health support devices also influenced negative assessments. In relation to the hospital infrastructure, it was mentioned that “the bed there isn’t very comfortable” (C14), the food was “different” (C15) from the food at home, “it didn’t look good” (C7), and there were “few options” (C15).

Accepting the food offered, even though she thought it was “very different”, was the strategy used by C3. For her, eating the hospital food was a habit change she had to make to “feel a little better”. To overcome the difficulty of eating the food offered at the hospital, children asked for options of dishes they liked and accepted food sent by other family members.

The medical devices used, such as a nasal catheter, were highlighted by C7 and C6 as causing discomfort due to dryness of the upper airways. Peripheral venous access caused pain and fear for C13, limiting C3’s and C4’s activities, since “it became more difficult because I like to color with this hand [right hand]” (C4). In order to cope with the discomfort and sadness they felt from having a nasal catheter, children relied on their primary caregivers: C6 “stayed close to my mother”; C1 reports on the support received, saying that his mother “kept getting water” for him; and C3 says that his mother “told me I would get better”.

The care routine established during hospitalization, disregarding children’s rest periods, was one aspect mentioned. C7 expressed that “night would come, and I had to get an injection! And I was already trying to fall asleep”. The severity of C7’s clinical condition, which included motor paralysis and swelling of the face and tongue, resulting in difficulty speaking and moving, contributed to her predominantly negative self-assessment, marked by stress and nervousness: “I wanted to speak so my mother would understand me, but when I tried to say a word, an ‘iiiii’ would come out immediately. So, I would get stressed” (C7).

Children identified that adopting self-care practices helped them feel better and reduce negative feelings. Children reported that body care and hygiene, such as leaving bed to take care of themselves (C1, C2, C3, C7), contributed to their well-being and helped alleviate negative feelings during hospitalization. “Drinking water” and “going to the bathroom” were the strategies used by C1 when feeling sadness and anger.

Adhering to the prescribed treatment was also a coping strategy reported by children. For C2, “medicine, injections... things” were important for him to feel better in the hospital. Two children cited their positive outlook on the illness as a coping strategy that allowed them to visualize positive outcomes from their hospitalization. C13, upon learning he had COVID-19, thought he would “get better quickly” and was not worried, as he perceived himself as “very strong!”. Being strong was also present in C7’s statements, and this self-perception was seen as a resource for “feeling better and having more energy”, allowing these children to make hospitalization more acceptable and manageable.

Only C2 provided information that allowed us to characterize his assessment of hospitalization as predominantly positive. The child assessed that his hospitalization was “good”, that he felt “happy” most of the time because, in the hospital, he “ate, drank, played games, lay down, and my mother would get water for me”.

Resignation was another strategy used by children during their hospitalization. At times, they chose to do nothing about their illness or hospitalization. C4 says she did “nothing… just kept thinking!” when she felt like going back “home and going to my grandma’s house”. And C7 said she “stayed quiet and thought” when she missed her family.

When describing their experience of hospitalization, a series of unpleasant experiences became evident. Although many of these were associated with healthcare professionals’ actions, professionals were identified by them as important resources for conversation (C3, C7), “feeling happy” (C3), “feeling more comfortable” (C3), or believing that “I could leave soon” (C7).

Children who perceived themselves as strong and comfortable in the face of hospitalization reported coping strategies that included a positive outlook and acceptance of support from healthcare professionals.

### Appreciation and coping strategies of primary caregivers regarding the hospitalization of children due to COVID-19

Caregivers’ assessments were influenced by hospital settings, the care received, and children’s health condition. Unlike the children, their strategies were primarily centered on interpersonal relationships to cope with the separation from family during hospitalization.

It is noteworthy that all primary caregivers reported fear or thoughts of death upon learning of a child’s hospitalization due to confirmed or suspected COVID-19. They stated that they were “afraid of losing her” (M7), thinking that their child “was going to pass away” (V14), or feeling “very afraid... of not being able to save her life, of there being no way out” (M15). It was observed that fear and thoughts of death were associated with access to information about the high mortality rate from COVID-19, coming from both reports from acquaintances and from media outlets. V14 recounts that “you see the TV station emphasizing it, people emphasizing it... and so many people dying and the girl still gets it. You think, ‘Oh, she’s going to die’”. In this regard, all caregivers who had lost friends or family members to COVID-19 before their children’s hospitalization presented predominantly negative assessments. M10, for instance, faced the loss of two uncles and her mother, which influenced her experience during her son’s hospitalization, stating that “the world was collapsing. It collapsed”. During this period, M10 suffered from depression and was not allowed to remain as the child’s companion in the hospital.

V1, a nurse, reported that her experience in a field hospital during the pandemic influenced her negative assessment: “[You] already know that behavior, right? What’s going to happen. The feeling that a person isn’t going to come back” (V1).

Identifying an improvement in children’s health condition was an important assessment that allowed primary caregivers to rework their coping strategies and build more positive perspectives: “he started to get better, and I think I wasn’t so scared anymore” (M13); “seeing her develop was one of the things that made me feel more confident” (M4). In contrast, M8 could not see any improvement in her son’s health, and he died due to the severity of his condition. Therefore, her coping strategies were different: she reported that “she didn’t sleep until C8 took his last medication, she brought him food, juice, a biscuit... things he liked to eat” (M8).

Another factor that triggered negative assessments (V1, M3, M4, M5, M6, M11, M12 and M13) was a child’s isolation during hospitalization. This is because it was a factor responsible for the feeling of “fear of not being able to cope” with caring for her daughter, in addition to feelings of loneliness and lack of support: “The hardest part was that I had to be alone a lot, and I needed help, you know?” (M11).

Isolation also prevented some parents and family members from entering the hospital during hospitalization and/or sharing in the care of their children during this time. M5 reports that “his [C5’s] father was desperate. He didn’t know whether to stay there [at the hospital], or stay here [at home], that he had other children to take care of”. M6 reports that the moments she wasn’t with her son were agonizing “[...] even though his [C6’s] father was with him, we couldn’t even rest” (M6).

In order to reduce loneliness, “video calls” (M4), audio conversations, and social media were used. For M7, “conversations” with her family played an important supporting role in coping with her daughter’s hospitalization. M13 also mentions “talking a lot” with her son as a strategy for distraction for both herself and the child. M6 and M11 said that “loving” (M6, M11) and “wanting the best for” (M11) the child were sources of inner strength that functioned as coping strategies.

Spirituality and faith in God were also resources for coping with the stressful situation, stating that “the only person who gives us strength is God” (V14) and “Our Lady of Aparecida” (M10). The participants also reported “asking God” to increase oxygen saturation (M2) by suspending oxygen therapy (M5) and by overcoming a child’s hospitalization (M4).

Predominantly positive assessments were identified in the reports of M2, M9, and M15. The reasons given for this assessment were that “the hospitals were well-prepared” (M9). Her son was “very well cared for” (M9, M15), with attention and affection (M2), and that he found “all the support in the hospital” (M9). M15 reports that “they treated my son as if he were their own son”. Professionals said “prayers” for each patient, and this made her feel more “calm and secure” (M15). V1, M2, M3, M5, M7, M9, and M14 cite “conversations with professionals” as a resource that “comforts” (M14) and left them feeling “very confident” (M2), “calmer” (M7), “grateful, with self-esteem” (V1), and supported.

On the other hand, the lack of information about children’s health status and the professionals’ negligence regarding biosafety practices contributed to negative assessments. M11 reported being informed about the COVID-19 confirmation only on the last day of C11’s hospitalization, while M13, even though present, did not receive sufficient clarification to understand her son’s condition: “He just kept having tests, and nothing effective helped him get better”. “I needed to know where I stood” (M13). Due to the lack of information, M4 characterized C4’s hospitalization as “a terrible experience”. Situations of this nature were decisive in leading M4 and M6 to contact the police as a means to be heard within the institution.

Regarding biosafety practices, M8 stands out, mentioning a lack of care from professionals regarding the risk of virus transmission: “There were professionals who sometimes cleaned the room; there were professionals who didn’t clean it at all”.

Comparing C6’s health situation to that of other hospitalized children was a coping strategy used by M6. She also noted that being with her child (M2), not letting her children see her distressed (M3), and relying on the support of others to care for the hospitalized child (V1) and other children (M13) were listed as key coping strategies.

## DISCUSSION

From the perspective of the family resilience model, unexpected or unplanned events can be stressful and have the potential to compromise family functioning^(8)^. The use of this framework in this investigation contributed to understanding children’s and primary caregivers’ perspectives, as well as their respective strategies for coping with a child’s hospitalization due to COVID-19.

It is known that hospitalization in childhood means deprivation of children’s routine^(12)^ and experiencing procedures that trigger pain and negative feelings such as fear, sadness, anxiety, and anger^(4)^. The findings are similar to those of this study with regard to the feelings experienced^(13)^, but the hospitalization experience was marked by specific characteristics of COVID-19, such as its high transmissibility, lethality and uncertainties about the disease, which influenced experiences related to social isolation and diagnosis severity.

Concerning this last aspect, the intense and widespread dissemination of information during the pandemic, described by the World Health Organization as an infodemic^(14)^, contributes to understanding the reasons for the negative and intense feelings expressed by participants. Children associated the pandemic context with images of death, anxiety, and sadness due to SARS-CoV-2 infection^(15)^, indicating the need to discuss the accountability of media outlets and content producers regarding the dissemination of sensationalist information and the exposure of families’ suffering. The infodemic can foster negative and stressful thoughts associated with mental disorders, requiring action from health surveillance teams.

At the same time, scientific information with robust evidence supports professional practices, guiding actions and mitigating negative assessments by identifying coping strategies that can be used depending on the health condition and the context experienced. Just as a child’s health situation during hospitalization evolves, so do assessments and coping strategies, which require further elaboration and individualized actions.

In addition to access to information, lifestyle at home also significantly influenced the hospitalization experience. While some children, like C2, valued aspects such as food and rest, others reported negatively about hospitalization due to the food and the monotony of the routine. This highlights the need to consider each hospitalization experience as unique, since previous experiences influence coping mechanisms. It is up to healthcare professionals to develop an individualized care plan, aligned with coping strategies consistent with each individual’s experience.

As for the use of electronic devices, children reported using cell phones and television as coping strategies during hospitalization for COVID-19. These coping strategies are similar to behaviors adopted more broadly by children during the pandemic^(16)^ due to being hospitalized. Although participants acknowledged benefits such as audio calls, video calls, and increased happiness, these practices were not institutionalized but rather carried out using electronic devices belonging to their own families. In a country like Brazil, with significant social inequality, where not everyone has access to electronic devices and quality internet, the institutional incorporation of Information and Communication Technologies, along with established norms for their use in hospital settings, is recommended.

Hence, although the study sought to include hospitalized children in different types of institutions, approximately 80% of participants were treated in private hospitals. This profile possibly influenced both the perceptions and coping strategies reported, as well as the conditions of access to technologies that enabled data collection via video call. This context differs from the reality of public institutions, frequently marked by structural and digital restrictions, especially evident during the COVID-19 pandemic^(17)^.

All the children interviewed reported coping strategies in the face of their negative assessments, with the presence of a companion being one of the main ones, as it fosters a feeling of security. According to the Statute of the Child and Adolescent^(18)^, this right is guaranteed to minors under 18 years of age. Studies carried out in other contexts indicate that the presence of a companion is fundamental for children to become familiar with and adapt to hospital settings and routine^(19)^, in addition to strengthening the family bond, allowing them to perceive the positive experience^(13)^.

It is known that play is a way in which children represent the real world, integrating cognitive, affective and interpersonal skills that favor their adjustment during hospitalization^(20)^. In the present study, play emerged as a coping strategy, aligning with findings from other research that point to play as a leisure resource to alleviate the focus on illness and treatment^(21)^.

Primary caregivers, as in other studies^(22)^, demonstrated satisfaction with nursing care, highlighting professionals’ care, attention, and affection, as well as gestures such as prayers with children, as elements that contributed to positive assessments. However, the lack of information provided by the health team generated insecurity in some caregivers, which was also observed in other studies^(22)^. In more critical cases, the lack of effective communication led family members to call the police, an event explained by communication breakdowns between professionals and caregivers, which are essential for building a relationship of trust and partnership in care.

In their reports, they also mentioned love for their children as a coping strategy. This finding is consistent with the literature, in which such a feeling is described as a source of unconditional love and lasting bonding^(23)^. Therefore, being with their children was listed as a positive factor during hospitalization and as a coping strategy that alleviated negative feelings. Conversely, M10 cannot be with C10 because she suffered from depression, which prevented her from staying at the hospital to accompany her son. In this context, the role of healthcare professionals in encouraging and facilitating virtual contact between the dyad is highlighted. A study with hospitalized children with chronic conditions showed that interactions with friends and family, through calls and cards, improved their mood and made children feel strong enough to face their hospitalization^(24)^.

The search for spirituality and religiosity was an important coping strategy used by caregivers to minimize feelings of fear. Religiosity represents connections with the Divine or the Sacred, capable of boosting faith^(25)^, generating hope in child caregivers, as well as feelings of tranquility, peace and strength to overcome and adapt to the situation of a child’s hospitalization^(26)^. During the pandemic, spirituality and religiosity stood out as effective coping strategies in reducing stress, anxiety, and depression, as well as increasing resilience^(27)^. Similar to this study, others conducted with families of hospitalized children show that spirituality involves confidence in the improvement and healing of their children, contributing to finding comfort in the face of the anguish that permeates childhood hospitalization^(28)^.

Social support was also listed as a coping strategy used by caregivers of hospitalized children. According to the theoretical framework adopted in this study, social support is defined as all the people or institutions that the family accesses as a protective factor in the face of an adverse situation^(8)^. In this way, the support network, made up of friends and family, helped to minimize loneliness in the face of social distancing, through calls, messages, prayers, childcare, and financial assistance.

### Study limitations

The main limitation of this study relates to the time interval between hospitalization and the interview, which may have influenced participants’ memory. Furthermore, because it was conducted during a specific period of the pandemic, changes in context and the emergence of new variants may impact assessments and coping strategies.

### Contributions to nursing, health, or public policy

The findings of this study, aligned with recent research^(21,29)^, highlight the complexity of the experiences lived by children hospitalized for COVID-19 and their caregivers, emphasizing the need for more sensitive, integrated, and individualized nursing practices. This research contributes to the field by offering concrete support for improving quality of care, highlighting the role of nursing in identifying and strengthening family support networks, in the systematic provision of spiritual support, and in the incorporation of Information and Communication Technologies in care. These strategies promote emotional well-being and child development continuity during prolonged hospitalizations.

Thus, this study broadens the understanding of childand family-centered care, reaffirming nursing’s commitment to expanded and humanized approaches.

## FINAL CONSIDERATIONS

Most children reported negative assessments associated with feelings such as fear, sadness, boredom, homesickness, restrictions of isolation, institutional feeding, and physical limitations. As coping strategies, they highlighted the use of electronic devices, the presence of a caregiver, play, and personal care. Caregivers with a stronger bond with the healthcare team had more positive assessments and sought support from professionals. Those who were dissatisfied resorted to spirituality, love for their children, denial of hospitalization, and virtual support from family members. The findings reinforce the need to offer systematic and sensitive care tailored to the specificities of childhood illness in infectious contexts, including the use of electronic devices to promote play, socialization, and education. The role of nurses in effective communication, building relationships, strengthening the support network, and promoting play as part of care is also highlighted.

## Data Availability

The research data are available only upon request.
