# Supplementary material for: Appreciation and coping strategies adopted by children hospitalized for COVID-19 and their primary caregivers
Source: Rev Bras Enferm. 2026 Jul 31;79:e20250265. doi: 10.1590/0034-7167-2025-0265 (PMC13435859; doi:10.1590/0034-7167-2025-0265)
Supplement: Translation

# Translation: Apreciação e estratégias de *coping* adotadas por crianças hospitalizadas por COVID-19 e suas cuidadoras primárias

**DOI:** 10.1590/0034-7167-2025-0265pt

**Authors:** Bárbara Radieddine Guimarães, Juliana Barony da Silva, Nayara Luiza Henriques, Ana Clara Gomes Andrade, Luiza Araujo dos Santos, Paulo Roberto Lima Falcão do Vale, Elysângela Dittz Duarte

**Affiliations:** I Universidade Federal de Minas Gerais. Belo Horizonte, Minas Gerais, Brasil; II Universidade Federal do Recôncavo da Bahia. Cruz das Almas, Bahia, Brasil

**Keywords:** COVID-19, Enfermagem, Criança, Cuidadores, Habilidades de Enfrentamento.

## Abstract

**Objetivos:**

analisar a apreciação e as estratégias de *coping* de crianças hospitalizadas por COVID-19 e de suas cuidadoras primárias durante o processo de adoecimento e internação.

**Métodos:**

estudo qualitativo e exploratório, baseado no modelo de McCubbin &, McCubbin (1993), com 25 participantes: dez crianças hospitalizadas por COVID-19 e 15 cuidadoras primárias. Utilizou-se entrevista semiestruturada cujos dados foram submetidos à análise temática reflexiva.

**Resultados:**

as crianças relataram apreciação predominantemente negativa da hospitalização, marcada por medo, saudade, dor e desconforto com a rotina hospitalar. Suas estratégias de *coping* incluíram brincadeiras, dispositivos eletrônicos, higiene e adesão ao tratamento. Cuidadores também expressaram avaliações negativas relacionadas ao medo de morte, isolamento e angústia. Suas estratégias envolveram espiritualidade, religiosidade, amor pelos filhos e apoio de profissionais e familiares.

**Considerações Finais:**

as estratégias de *coping* diferiram entre crianças e cuidadores, ressaltando a importância de cuidados sensíveis às necessidades da díade.

## INTRODUÇÃO

A pandemia de COVID-19, iniciada em 2019, teve repercussão mundial nos âmbitos sociais, econômicos e da saúde da população, causando milhares de mortes. Crianças entre 0 e 14 anos representaram entre 1,68% e 16,34% dos casos entre dezembro de 2019 e julho de 2023^(1)^. Dados epidemiológicos têm indicado desfechos de saúde menos graves para essa população infantil em comparação à população adulta^(2)^; no entanto, há registros de manifestações graves e necessidade de hospitalizações pediátricas por essa causa^(3)^.

A hospitalização é um evento estressante para toda a família, provocando mudanças na rotina e no bem-estar dos membros. Quando a internação é de crianças, a dependência física e emocional existente nesse grupo traz demandas adicionais aos cuidadores e familiares que referem desgaste relacionado à alteração da rotina, aumento dos gastos e privação de sono^(4)^. Além disso, sentimentos de angústia, ansiedade, perda da privacidade e preocupação têm sido identificados em crianças e seus cuidadores em situação de internação^(4,5)^. A dor e o medo, por sua vez, são vivências referidas somente pelas crianças^(4,5)^.

Em contexto de pandemia de COVID-19, a experiência de internação teve particularidades, como a possibilidade de contaminação de familiares e a estigmatização da doença^(6,7)^, especialmente antes da disponibilidade de vacinas e do reconhecimento da maior gravidade dos casos que demandaram hospitalização^(6)^. Esses aspectos foram identificados como contribuintes para acentuar a vulnerabilidade das crianças, gerar preocupação e estresse^(6)^, e agravar os quadros de ansiedade e depressão em toda população^(7)^.

Mesmo com aspectos comuns, a forma de lidar com situações estressoras, a exemplo da hospitalização de uma criança por COVID-19, varia de acordo com a dinâmica familiar e com as adaptações feitas por cada membro e pela família como um todo^(8)^. Sendo assim, a apreciação que as famílias fazem sobre um mesmo evento estressor, neste estudo, o adoecimento e hospitalização da criança, pode ser variável entre as famílias e os seus integrantes, assim como a utilização de recursos para se adaptar a essa situação^(8)^.

Neste estudo, adotaram-se o Modelo teórico de Resiliência, Stress, Ajustamento e Adaptação Familiar^(8)^ e sua definição de apreciação e *coping*. A apreciação é definida como a maneira com que a família e/ou seus membros, individualmente, percebem e sentem o evento estressor, sua gravidade e os significados das mudanças ocorridas na dinâmica e no cotidiano familiar^(8)^. O *coping*, por sua vez, diz respeito às estratégias, esforços e comportamentos adotados pela família e/ou seus membros, individualmente, para manter ou restabelecer o equilíbrio entre as demandas e os recursos familiares, reduzindo a severidade do estressor, neste estudo, a internação por COVID-19 e seus desafios^(8)^.

Portanto, a apreciação familiar da situação vivenciada e as estratégias de *coping* estão interrelacionadas e podem ser modificadas à medida que as famílias se tornam mais vulneráveis por experimentarem um acúmulo de demandas quando um membro fica doente^(8)^.

Destaca-se que, a depender da apreciação que cada um dos membros faz sobre a situação vivenciada, pode demandar esforços diferenciados para o enfrentamento da situação. Assim, admite-se a importância de explorar, sob a perspectiva das crianças e das cuidadoras primárias, as estratégias de *coping* utilizadas durante a hospitalização por COVID-19.

## OBJETIVOS

Analisar a apreciação e as estratégias de *coping* de crianças hospitalizadas por COVID-19 e de suas cuidadoras primárias durante o processo de adoecimento e internação.

## MÉTODOS

### Aspectos éticos

A pesquisa foi aprovada pelo Comitê de Ética em Pesquisa da Universidade Federal de Minas Gerais e pela Secretaria Municipal de Saúde de Belo Horizonte, conforme as Resoluções n^o^ 466/12 e n^o^ 580/18 do Conselho Nacional de Saúde. A participação foi autorizada mediante assinatura do Termo de Consentimento Livre e Esclarecido, pelas cuidadoras, e do Termo de Assentimento Livre e Esclarecido, pelas crianças, enviados por meio eletrônico ou correio.

### Referencial teórico-metodológico

A pesquisa foi orientada pelo Modelo de Resiliência, Stress, Ajustamento e Adaptação Familiar^(8)^, que permitiu compreender a hospitalização da criança com COVID-19 como um momento de estresse vivido pela família, no qual estratégias podem ser adotadas para a construção de uma perspectiva mais positiva.

### Tipo de estudo

Trata-se de estudo qualitativo do tipo exploratório, cuja elaboração atendeu às recomendações do guia *Consolidated criteria for Reporting Qualitative research* ^(9)^.

### Procedimentos metodológicos

As famílias do estudo foram identificadas a partir de um banco de dados, disponibilizado pela vigilância epidemiológica da prefeitura de Belo Horizonte, Minas Gerais. Esse banco reunia informações provenientes das fichas de notificação de 83 crianças que haviam sido hospitalizadas por COVID-19.

A partir desses dados, foram realizados os primeiros contatos telefônicos com as famílias, a fim de apresentar os objetivos do estudo e convidar o cuidador primário a participar. Mediante o aceite, foi agendado o melhor dia e horário, conforme disponibilidade do familiar.

Destaca-se que, entre as 83 crianças do banco de dados fornecido, 44 não possuíam informações para contato ou o número era inexistente; dez não atenderam; sete responsáveis relataram que a criança não foi hospitalizada por suspeita ou COVID-19 confirmado; e sete recusaram participar da pesquisa.

Critérios de inclusão compreenderam cuidadores principais que residiam no mesmo domicílio que a criança e crianças de 6 a 12 anos - pela capacidade de compreensão e reflexão^(10)^ - hospitalizadas por suspeita ou comprovação de infecção por SARS-CoV-2. Critérios de exclusão incluíram cuidadores com limitações de comunicação ou alterações psicológicas/psiquiátricas que inviabilizassem a compreensão dos procedimentos investigativos.

### Cenário do estudo

O presente estudo foi realizado no município de Belo Horizonte, Minas Gerais. A identificação dos participantes foi realizada a partir de um banco de dados disponibilizado pela vigilância epidemiológica da prefeitura.

O desenvolvimento do estudo ocorreu por meio de entrevistas por videochamadas (*WhatsApp^®^* ou *Google Meet^®^*), respeitando as condições impostas pelo contexto sanitário do período.

### Fonte de dados

Os participantes da pesquisa foram dez díades compostas por crianças, que foram hospitalizadas pela COVID-19 e suas respectivas cuidadoras primárias, além de cinco cuidadoras cujas crianças não puderam participar pelos seguintes motivos: duas adoeceram no dia da entrevista; uma se recusou participar; uma se ausentou no momento da entrevista; e uma faleceu após a hospitalização.

### Coleta e organização dos dados

Para a coleta de dados, utilizou-se um questionário sociodemográfico aplicado às cuidadoras primárias, além de entrevistas semiestruturadas conduzidas com roteiros elaborados com base no modelo de McCubbin & McCubbin^(8)^, que foram distintos para crianças e cuidadoras. As questões se nortearam pelos desafios vivenciados e pelas estratégias de *coping* durante a hospitalização, incluindo perguntas como “Como foi ficar no hospital um tempo por causa do coronavírus?” (criança) e “Me conta como foi para você receber a notícia de que o(a) [nome] precisaria ficar hospitalizado(a) por COVID?” (cuidadora).

As entrevistas foram conduzidas separadamente, orientando-se que cuidadoras e crianças permanecessem sozinhas para garantir a fidedignidade dos dados e a liberdade de expressão. Apenas em duas situações a criança esteve no mesmo ambiente da cuidadora.

A coleta foi realizada entre outubro de 2021 e janeiro de 2022, com o intervalo entre a hospitalização e a entrevista variando de cinco meses a um ano e meio. As entrevistas foram audiogravadas e tiveram duração média de 34 minutos. No decorrer das entrevistas, foi produzido um diário de campo, cujos registros foram utilizados pela pesquisadora para apoiar especialmente a análise das informações.

Os contatos foram realizados por uma das autoras, enfermeira com experiência em pesquisa e cuidado infantil, no contexto de seu mestrado.

### Análise dos dados

As entrevistas foram transcritas, e os dados qualitativos foram submetidos à análise temática reflexiva^(11)^, guiada pelo Modelo de Resiliência, Stress, Ajustamento e Adaptação Familiar (McCubbin & McCubbin, 1993)^(8)^. O *software* MAXQDA^®^ foi utilizado para gerenciar, codificar e explorar os dados. Os códigos foram construídos a partir do referencial utilizado e de aspectos comuns entre as falas das crianças e das cuidadoras que poderiam ajudar a responder à pergunta de pesquisa.

Inicialmente, seis entrevistas (três entrevistas de crianças e três das cuidadoras primárias) foram codificadas por duas pesquisadoras independentes. Em seguida, os códigos e eventuais dúvidas ou divergências foram discutidos com uma terceira autora. Durante essa etapa de consenso da codificação, foi alcançado um índice Kappa maior que 0,75 para a concordância intercodificadores. Concluída a codificação das entrevistas, foi construída uma matriz analítica contendo a síntese de cada participante com as informações de cada um dos códigos. A matriz permitiu dar visibilidade às experiências individuais e identificar os padrões existentes nos dados produzidos.

Esse processo resultou na definição dos seguintes temas: “Apreciação e estratégias de *coping* adotadas por crianças hospitalizadas por COVID-19” e “Apreciação e estratégias de *coping* das cuidadoras primárias sobre a hospitalização das crianças por COVID-19”.

## RESULTADOS

Foram entrevistados 25 participantes, sendo 15 cuidadoras primárias e dez crianças hospitalizadas por COVID-19, formando dez díades. Não compuseram díades M8, M9, M10, M11 e M12. Entre as cuidadoras, 13 eram mães, e duas, avós, com idades entre 25 e 49 anos (mediana = 42). A maioria era casada (n=9), tinha alguma religião (n=13), autodeclarava-se parda (n=9), exercia trabalho remunerado (n=10) e possuía ensino superior incompleto (n=6). As dez crianças tinham entre 6 e 11 anos (mediana = 6), sendo a maioria do sexo masculino (n=6), internadas em hospitais privados (n=8), com mediana de sete dias de internação (2-96 dias). Quatro tinham comorbidades como asma (n=3) e crises convulsivas (n=1).

Quanto ao contexto pandêmico, 11 internações ocorreram com ocupação de Unidade de Terapia Intensiva acima de 70%, e quatro, durante períodos de maior restrição social. Todas as internações ocorreram antes da disponibilização da vacina contra a COVID-19 para o grupo pediátrico.

Para melhorar a apresentação dos resultados, esta sessão foi organizada em dois temas: “Apreciação e estratégias de *coping* adotadas por crianças hospitalizadas por COVID-19” e “Apreciação e estratégias de *coping* das cuidadoras primárias sobre a hospitalização das crianças por COVID-19”. Identificaram-se padrões de experiências predominantemente positivas, associadas a sentimentos como alegria, confiança e tranquilidade, e predominantemente negativas, marcadas por insatisfações, sentimentos difíceis e eventos estressores vivenciados durante a hospitalização.

### Apreciação e estratégias de *coping* adotadas por crianças hospitalizadas por COVID-19

A apreciação das crianças sobre a hospitalização por COVID-19 foi expressa por seus sentimentos e pelos significados atribuídos ao adoecimento, aos sintomas e aos cuidados recebidos. A análise de dados revelou que as estratégias de *coping* adotadas por elas foram diversas e relacionadas às diferentes formas de apreciação referidas.

O medo foi um sentimento referido pelas crianças como responsável por uma apreciação predominantemente negativa, como apresentado por C2, ao contar ter pensado “que eu ia morrer!”. C3 refere ter sentido medo por ouvir relatos de morte de parentes: “parente meu morreu por causa do COVID, então você ficava com medo, né?”.

Além do medo, o “isolamento, a monotonia e a falta de interação” no hospital foram motivos de apreciação predominantemente negativa das crianças. C3, C4, C7 e C13 falam sobre isso ao relatarem que, durante sua hospitalização, não podiam fazer nada, porque ficar “quietinha era a única coisa que eu tinha pra fazer” (C7). Tinham “que ficar toda hora olhando para a mesma vista [...] tinha que ficar só no quarto” (C4).

Também contribuiu para apreciações negativas o fato de “não estar na sua [própria] casa” (C3). As crianças relatam sentimentos como a saudade de “ir para a casa da minha vó” (C3) e “da minha família” (C7), e tristeza de saber que, “se você está no hospital, você sabe que você não está bem” (C3). Algumas crianças relatam a sensação de que o tempo de hospitalização foi muito longo: “eu fiquei internado 4 mil dias lá!” (C6); “o cheiro do hospital era até bom, mas ficar internada mil anos depois, não” (C4). Para essas apreciações, a manutenção do contato com os familiares através de celulares foi uma estratégia de *coping*. Dispositivos como televisores e *tablets* foram utilizados para reduzir saudade (C1, C6), medo (C2, C6, C13), tristeza (C6), tédio (C7), e para “passar o tempo” (C3) e “distrair” (C1). O uso de brincadeiras também foi uma estratégia utilizada. As crianças brincavam com seus “joguinhos” (C2, C13), além de “animais e bonequinhos” (C13) para “distrair um pouco” (C13) e para sentir menos “medo” (C2, 13) e “tristeza” (C2) no hospital. Além das brincadeiras, passar o tempo com seus acompanhantes e aceitar o apoio deles também foram vistos como estratégias para manejar as tensões relacionadas à hospitalização.

Aspectos da estrutura hospitalar, dos serviços e dos dispositivos de suporte em saúde também influenciaram apreciações negativas. Sobre a infraestrutura hospitalar, foi referido que “a cama de lá não é muito confortável” (C14), a comida era “diferente” (C15) da comida de suas casas, “não estava com uma cara boa” (C7) e tinha “poucas opções” (C15).

Aceitar a alimentação oferecida, mesmo achando que ela era “muito diferente”, foi a estratégia utilizada por C3. Para ela, comer a comida do hospital foi uma mudança de hábito que teve que fazer para “ficar um pouco melhor”. Para superar a dificuldade de comer os alimentos oferecidos no hospital, as crianças pediram opções de pratos de que gostavam e aceitaram comidas enviadas por outros familiares.

Os dispositivos de saúde utilizados, como o cateter nasal, foram destacados por C7 e C6 como geradores de desconforto devido ao ressecamento de vias aéreas superiores. Os acessos venosos periféricos provocaram dor e medo para C13, limitando as atividades de C3 e C4, visto que “ficou mais difícil porque eu gosto de colorir com essa mão [mão direita]” (C4). A fim de lidar com o desconforto e a tristeza que sentiam por estar com um cateter nasal, as crianças contavam com suas cuidadoras primárias: C6 “ficava pertinho da minha mãe”; C1 relata sobre o apoio recebido, dizendo que sua mãe “ficava pegando água” para ele; e C3 conta que sua mãe “falava que eu iria melhorar”.

A rotina de cuidados estabelecida durante a internação, desconsiderando os momentos de repouso das crianças, foi um aspecto referido. C7 expressa que “chegava à noite, eu tinha que tomar uma injeção! E eu já estava querendo pegar no sono”. A gravidade do quadro clínico de C7, que incluiu paralisia motora e inchaço da face e língua, resultando em dificuldade para falar e se mover, contribuiu para sua apreciação predominantemente negativa, marcada por estresse e nervosismo: “eu queria falar para minha mãe me entender, mas quando eu ia falar alguma palavra, saia um ‘iiiii’ na hora. Então, eu ficava estressada” (C7).

As crianças identificaram que a adoção de cuidados as ajudava a se sentir melhor e diminuir sentimentos negativos. As crianças referiram que cuidados com o corpo e a higiene, como sair da cama para se cuidar (C1, C2, C3, C7), contribuíam para seu bem-estar e ajudavam a amenizar sentimentos negativos durante a hospitalização. “Bebia água” e “ia no banheiro” foram as estratégias usadas por C1 quando sentia tristeza e raiva.

Aderir ao tratamento proposto também foi uma estratégia de *coping* relatada pelas crianças. Para C2, “remédio, injeção... coisas” foram importantes para que ele ficasse bem no hospital. Duas crianças citam o seu olhar positivo diante da situação de adoecimento como uma estratégia de *coping* que as permitiram visualizar desfechos positivos de sua hospitalização. C13, ao saber que estava com COVID-19, pensou que “ia melhorar rápido” e não ficou preocupado, uma vez que se percebia “muito forte!”. Ser forte também esteve presente nas falas de C7, e essa autopercepção foi vista como um recurso para “se sentir melhor e com mais energia”, permitindo que essas crianças tornassem a hospitalização mais aceitável e manejável.

Somente C2 apresentou informações que permitiram caracterizar a sua apreciação sobre a hospitalização como predominantemente positiva. A criança avalia que sua hospitalização foi “legal”, que sentia “alegria” na maior parte do tempo porque, no hospital, ele “comia, bebia, ficava jogando, ficava deitado, aí minha mãe ficava pegando água pra mim”.

A resignação foi outra estratégia utilizada pelas crianças durante sua hospitalização. Por vezes, elas optaram por não fazer nada em relação ao seu processo de adoecimento ou hospitalização. C4 conta que não fazia “nada... só ficava pensando!” quando sentia vontade de voltar para “casa e de ir para a casa da minha vó”. E C7 dizia que “ficava quietinha e pensava” quando sentia falta da sua família.

Ao descreverem sua apreciação sobre a internação, ficou evidente um conjunto de experiências desagradáveis. Embora muitas delas tenham sido associadas a ações dos profissionais de saúde, os profissionais foram identificados por elas como importantes recursos para conversar (C3, C7), “se sentir feliz” (C3), “se sentir mais confortável” (C3) ou acreditar que “eu poderia sair logo” (C7).

Crianças que se reconheciam fortes e confortáveis diante da internação reportaram estratégias de *coping* que incluíam uma perspectiva positiva e aceitação do apoio dos profissionais de saúde.

### Apreciação e estratégias de *coping* das cuidadoras primárias sobre a hospitalização das crianças por COVID-19

As apreciações das cuidadoras foram influenciadas pelo ambiente hospitalar, pelo cuidado recebido e pela condição de saúde da criança. Ao contrário das crianças, suas estratégias foram centradas, principalmente, nas relações interpessoais para lidar com a distância da família durante a hospitalização.

Destaca-se que todas as cuidadoras primárias referiram medo ou pensamentos de morte ao saber da hospitalização da criança por confirmação ou suspeita de COVID-19. Elas referem que ficaram “com medo de perder ela” (M7), ao pensar que sua criança “ia embora” (V14) ou ao sentir “muito medo... de não conseguir salvar a vida, de não ter jeito” (M15). Observou-se que o medo e o pensamento de morte estavam associados ao acesso a informações sobre a alta mortalidade por COVID-19, provenientes tanto de relatos de conhecidos quanto de veículos de comunicação. V14 conta que “você vê a emissora enfatizando, o povo enfatizando... e aquele tanto de gente morrendo e a menina ainda pega. Você fala: ‘Ah, vai morrer’”. Nesse sentido, todas as cuidadoras que haviam perdido amigos ou familiares por COVID-19 antes da hospitalização de suas crianças apresentaram apreciações predominantemente negativas. M10, por exemplo, enfrentou a perda de dois tios e da mãe, o que influenciou sua vivência durante a internação do filho, contando que “o mundo estava desabando. Desabou”. Nesse período, M10 teve depressão e não foi autorizada a permanecer como acompanhante da criança no hospital.

V1, enfermeira, relatou que sua experiência em um hospital de campanha durante a pandemia influenciou sua apreciação negativa: “[você] já sabe aquela conduta, né? O que vai acontecer. A sensação de que a pessoa não vai voltar” (V1).

Identificar melhora na condição de saúde da criança foi uma apreciação importante para que as cuidadoras primárias conseguissem reelaborar suas estratégias de *coping* e construir perspectivas mais positivas: “ele começou a melhorar, e eu acho que eu não tive mais medo assim” (M13); “ver o desenvolver dela foi uma das coisas que me fez ficar mais confiante” (M4). Em contrapartida, M8 não pôde perceber melhora na saúde do filho, que evoluiu a óbito devido à gravidade do quadro. Por isso, suas estratégias de *coping* foram distintas: ela informou que “não dormia enquanto C8 não tomava a última medicação, levava para ele comida, um suco, um biscoito... as coisas que ele gostava de comer” (M8).

Outro fator que desencadeou apreciações negativas (V1, M3, M4, M5, M6, M11, M12 e M13) foi o isolamento da criança durante a hospitalização. Isso porque ele foi um fator responsável pelo sentimento de “medo de não dar conta” de cuidar da sua filha, além do sentimento de solidão e a falta de apoio e suporte: “O mais difícil foi que eu tinha que ficar muito sozinha, e eu precisava de uma ajuda, né?” (M11).

O isolamento também impediu alguns pais e familiares de entrarem no hospital durante a hospitalização e/ou compartilhar do cuidado de suas crianças durante esse momento. M5 relata que “o pai dele [C5] ficou desesperado. Não sabia se ficava lá [no hospital], se ficava aqui [em casa], que tinha mais menino pra cuidar”. M6 relata que os momentos que ela não esteve com seu filho foram angustiantes “[...] por mais que o pai dele [C6] estava com ele, a gente não consegue nem descansar” (M6).

A fim de diminuir a solidão, foram realizadas as “chamadas de vídeo” (M4), conversas por áudio e redes sociais. Para M7, “conversas” com sua família tiveram um papel importante de apoio para o enfrentamento da hospitalização da filha. M13 também refere “conversar bastante” com o filho como uma estratégia para distração dela e da criança. M6 e M11 disseram que “amar” (M6, M11) e “querer o bem” (M11) da criança foram fontes de força interior que funcionaram como estratégias de *coping*.

A espiritualidade e a fé em Deus também foram recursos para lidar com a situação estressora, afirmando que a “única pessoa que dá força pra gente é Deus” (V14) e “Nossa Senhora Aparecida” (M10). As participantes também relataram “pedir a Deus” para aumentar a saturação de oxigênio (M2) pela suspensão da oxigenioterapia (M5) e pela superação da hospitalização da criança (M4).

Apreciações predominantemente positivas foram identificadas nos discursos de M2, M9 e M15. Os motivos referidos para esta apreciação foram que “os hospitais estavam bem-preparados” (M9). O filho foi “muito bem atendido” (M9, M15), com atenção e carinho (M2), e que encontrou “todo o apoio no hospital” (M9). M15 relata que “trataram o meu filho como se fosse filho deles”. Os profissionais faziam “orações” para cada paciente, e isso a deixava mais “tranquila e segura” (M15). V1, M2, M3, M5, M7, M9 e M14 citam as “conversas com profissionais” como um recurso que “conforta” (M14) e as deixaram “bem confiantes” (M2), “mais tranquilas” (M7), “agradecidas, com autoestima” (V1) e se sentindo apoiadas.

Por outro lado, a falta de informações sobre o estado de saúde da criança e o descuido dos profissionais com as práticas de biossegurança contribuíram para apreciações negativas. M11 relatou ter sido informada sobre a confirmação da COVID-19 apenas no último dia da internação de C11, enquanto M13, mesmo presente, não obteve esclarecimentos suficientes para compreender o quadro do filho: “Ele ficava só fazendo exame, e nada efetivo para que ele melhorasse. Eu precisava saber em que pé que estava” (M13). Por conta da falta de informações, M4 caracterizou a hospitalização de C4 como “uma péssima experiência”. Situações desta natureza foram determinantes para que M4 e M6 acionassem a polícia como um recurso para que fossem ouvidas dentro da instituição.

Sobre as práticas de biossegurança, destaca-se M8, que referiu falta de cuidado dos profissionais quanto ao risco de transmissão do vírus: “Tinha profissional que às vezes limpava o quarto; tinha profissional que não limpava”.

A comparação entre a situação de saúde de C6 e das outras crianças hospitalizadas foi uma estratégia de *coping* utilizada por M6. Também observou que estar junto de seu filho (M2), não deixar que suas crianças as vissem abaladas (M3) e contar com o apoio de outras pessoas para o cuidado da criança hospitalizada (V1) e de outros filhos (M13) foram elencadas.

## DISCUSSÃO

Sob a perspectiva do modelo de resiliência familiar, eventos inesperados ou não planejados podem ser estressores e têm o potencial de comprometer o funcionamento das famílias^(8)^. O uso desse referencial nesta investigação contribuiu para conhecer as perspectivas de crianças e cuidadoras primárias, bem como suas respectivas estratégias para lidar com a situação de hospitalização da criança por COVID-19.

Sabe-se que as situações de internação na infância significam privação de rotina das crianças^(12)^ e vivência de procedimentos que desencadeiam dor e sentimentos negativos como medo, tristeza, ansiedade e raiva^(4)^. Os achados se assemelham aos deste estudo no que diz respeito aos sentimentos vivenciados^(13)^, mas a experiência de internação foi marcada por características específicas da COVID-19, como sua alta transmissibilidade, letalidade e incertezas sobre a doença, o que influenciou vivências relacionadas ao isolamento social e à gravidade do diagnóstico.

Sobre este último aspecto, a intensa e ampla propagação de informações durante a pandemia, descrita pela Organização Mundial da Saúde como uma infodemia^(14)^, contribui para compreender os motivos de sentimentos negativos e intensos explicitados pelos participantes. As crianças associavam o contexto de pandemia com imagens de morte, ansiedade e tristeza, devido à infecção por SARS-CoV-2^(15)^, o que indica a necessidade de debater sobre a responsabilização dos veículos de comunicação e dos produtores de conteúdo sobre a divulgação de informações sensacionalistas e exposição do sofrimento das famílias. A infodemia pode favorecer pensamentos negativos e estressantes associados a transtornos mentais, exigindo ações das equipes de vigilância em saúde.

Ao mesmo tempo, informações científicas com evidências robustas subsidiam as práticas profissionais, de modo a orientar suas ações e atenuar as apreciações negativas por meio da identificação de estratégias de *coping* que podem ser utilizadas a depender da condição de saúde e do contexto vivenciado. Assim como a situação de saúde da criança durante a internação, a apreciação e as estratégias de *coping* são processuais, demandando novas elaborações e ações individualizadas.

Além do acesso à informação, o estilo de vida em seus lares também influenciou significativamente a experiência de internação. Enquanto algumas crianças, como C2, valorizaram aspectos como alimentação e repouso, outras relataram negativamente a hospitalização, devido à comida e à monotonia da rotina. Isso evidencia a necessidade de considerar cada experiência de internação como única, pois vivências prévias influenciam a forma de enfrentamento. Cabe ao profissional de saúde elaborar um plano de cuidado individualizado, alinhado às estratégias de *coping* coerentes com a vivência de cada indivíduo.

Sobre o uso de dispositivos eletrônicos, as crianças relataram usar celular e televisão como estratégias de *coping* durante a hospitalização por COVID-19. Essas estratégias de *coping* se assemelham a comportamentos adotados de forma mais ampla por crianças durante a pandemia^(16)^, por estarem hospitalizadas. Embora os participantes tenham reconhecido benefícios como chamadas de áudio, chamadas de vídeo e aumento da felicidade, essas práticas não foram institucionalizadas, mas realizadas a partir de aparelhos eletrônicos das próprias famílias. Em um país como o Brasil, de grande desigualdade social, em que nem todas as pessoas possuem acesso aos aparelhos eletrônicos e internet de qualidade, recomenda-se a incorporação institucional das Tecnologias de Informação e Comunicação associada às normas de uso dessas em instituições hospitalares.

Nesse sentido, embora o estudo tenha buscado incluir crianças hospitalizadas em diferentes tipos de instituições, aproximadamente 80% das participantes foram atendidas em hospitais privados. Esse perfil possivelmente influenciou tanto as percepções e estratégias de *coping* relatadas quanto as condições de acesso a tecnologias que viabilizaram a coleta de dados por videochamada. Tal contexto difere da realidade de instituições públicas, frequentemente marcadas por restrições estruturais e digitais, especialmente evidenciadas durante a pandemia de COVID-19^(17)^.

Todas as crianças entrevistadas relataram estratégias de *coping* diante de suas apreciações negativas, sendo a presença do acompanhante uma das principais, por favorecer o sentimento de segurança. Conforme o Estatuto da Criança e do Adolescente^(18)^, esse direito é garantido a menores de 18 anos. Estudos realizados em outros contextos apontam que a presença do acompanhante é fundamental para que a criança se familiarize e se adapte ao ambiente e à rotina hospitalar^(19)^, além de fortalecer o vínculo familiar, permitindo perceber a experiência positiva^(13)^.

Sabe-se que brincar é uma forma pela qual as crianças representam o mundo real, integrando competências cognitivas, afetivas e interpessoais que favorecem seu ajustamento durante a hospitalização^(20)^. No presente estudo, o brincar surgiu como estratégia de *coping*, alinhando-se a achados de outras pesquisas que apontam as brincadeiras como recurso de lazer para aliviar o foco na doença e no tratamento^(21)^.

As cuidadoras primárias, assim como em outros estudos^(22)^, demonstraram satisfação com o atendimento de enfermagem, destacando o cuidado, a atenção e o carinho dos profissionais, além de gestos como orações com as crianças, como elementos que contribuíram para apreciações positivas. No entanto, a insuficiência de informações fornecidas por parte da equipe de saúde gerou insegurança em algumas cuidadoras, o que também foi observado em outros estudos^(22)^. Em casos mais críticos, a falta de comunicação efetiva levou familiares a acionar a polícia, acontecimento que se explica pelos ruídos de comunicação entre profissionais e cuidadores, essencial para a construção de uma relação de confiança e parceria no cuidado.

Em seus relatos, também mencionaram o amor por suas crianças como uma estratégia de *coping*. Esse achado dialoga com a literatura, na qual tal sentimento é descrito como uma fonte de amor incondicional e de vínculo duradouro^(23)^. Por isso, estar com suas crianças foi elencada como um fator positivo da hospitalização e como uma estratégia de enfrentamento que amenizava os sentimentos negativos. Em contrapartida, M10 não pode estar junto de C10, por ter tido um quadro depressivo que a impossibilitou de permanecer no hospital como acompanhante do filho. Neste contexto, destaca-se o papel dos profissionais de saúde em incentivar e viabilizar o contato virtual entre a díade. Estudo com crianças com condições crônicas hospitalizadas evidenciou que interações com amigos e familiares, por meio de chamadas e cartões, melhoravam o humor e faziam com que a criança se sentisse forte para enfrentar sua hospitalização^(24)^.

A busca pela espiritualidade e pela religiosidade foi uma importante estratégia de *coping* utilizada pelas cuidadoras para minimizar o sentimento de medo. A religiosidade representa conexões com o Divino ou o Sagrado, capazes de impulsionar a fé^(25)^, gerar esperança em cuidadores de crianças, bem como sentimentos de tranquilidade, paz e força para superar e se adaptar à situação de internação de uma criança^(26)^. Durante a pandemia, a espiritualidade e a religiosidade se destacaram como estratégias de *coping* eficazes na redução do estresse, ansiedade e depressão, além de aumentarem a resiliência^(27)^. Assim como neste estudo, outros realizados com famílias de crianças hospitalizadas evidenciam que a espiritualidade envolve confiança na melhora e cura de seus filhos, contribuindo para encontrar conforto diante da angústia que permeia a hospitalização infantil^(28)^.

O apoio social foi também elencado como uma estratégia de *coping* utilizada pelas cuidadoras das crianças hospitalizadas. Segundo referencial teórico adotado neste estudo, o apoio social é definido como todas as pessoas ou instituições que a família acessa como fator de proteção diante da situação adversa^(8)^. Dessa forma, a rede de apoio, formada por amigos e familiares, ajudou a minimizar a solidão diante do distanciamento social, por meio de ligações, mensagens, orações, cuidado com outros filhos e auxílio financeiro.

### Limitações do estudo

A principal limitação deste estudo se refere ao intervalo de tempo entre a hospitalização e a entrevista, o que pode ter influenciado a memória dos participantes. Além disso, por ter sido realizado em um período específico da pandemia, as mudanças no contexto e o surgimento de novas variantes podem impactar as apreciações e estratégias de *coping*.

### Contribuições para a área da enfermagem, saúde ou políticas públicas

Os achados deste estudo, alinhados a pesquisas recentes^(21,29)^, evidenciam a complexidade das experiências vivenciadas por crianças hospitalizadas por COVID-19 e suas cuidadoras, ressaltando a necessidade de práticas de enfermagem mais sensíveis, integradas e individualizadas. A pesquisa contribui para a área ao oferecer subsídios concretos para a qualificação do cuidado, destacando o papel da enfermagem na identificação e fortalecimento das redes de apoio familiar, na oferta sistematizada de suporte espiritual, e na incorporação das Tecnologias de Informação e Comunicação na assistência. Tais estratégias favorecem o bem-estar emocional e a continuidade do desenvolvimento infantil durante hospitalizações prolongadas.

Assim, este estudo amplia a compreensão sobre o cuidado centrado na criança e na família, reafirmando o compromisso da enfermagem com abordagens ampliadas e humanizadas.

## CONSIDERAÇÕES FINAIS

A maioria das crianças relatou apreciações negativas associadas a sentimentos como medo, tristeza, tédio, saudade, restrições do isolamento, alimentação institucional e limitações físicas. Como estratégias de *coping*, destacaram o uso de dispositivos eletrônicos, a presença do acompanhante, o brincar e os cuidados pessoais. Cuidadoras com maior vínculo com a equipe de saúde tiveram apreciações mais positivas e buscaram apoio nos profissionais. Já aquelas insatisfeitas recorreram à espiritualidade, ao amor pelos filhos, à negação da hospitalização e ao apoio de familiares por meio virtual. Os achados reforçam a necessidade de oferecer um cuidado sistematizado e sensível às especificidades do adoecimento infantil em contextos infecciosos, com inclusão de dispositivos eletrônicos para favorecer o brincar, a socialização e a educação. Destaca-se também o papel do enfermeiro na comunicação efetiva, na formação de vínculos, no fortalecimento da rede de apoio e na promoção do brincar como parte do cuidado.

## Data Availability

Os dados de pesquisa só estão disponíveis mediante solicitação.
